# Sensitization of P2X3 receptors by cystathionine *β*-synthetase mediates persistent pain hypersensitivity in a rat model of lumbar disc herniation

**DOI:** 10.1186/s12990-015-0012-7

**Published:** 2015-03-20

**Authors:** Qianliang Wang, Hongyan Zhu, Kang Zou, Bo Yuan, You-Lang Zhou, Xinghong Jiang, Jun Yan, Guang-Yin Xu

**Affiliations:** Department of Orthopedics, the Second Affiliated Hospital of Soochow University, Suzhou, 215004 Peoples Republic of China; Jiangsu Key Laboratory of Translational Research and Therapy for Neuro-Psycho-Diseases, Institute of Neuroscience, Soochow University, Suzhou, 215123 Peoples Republic of China; Laboratory for Translational Pain Medicine, Institute of Neuroscience, Soochow University, 199 Ren-Ai Road, Suzhou, 215123 China

**Keywords:** Lumbar disc herniation, Dorsal root ganglion, Neuropathic pain, Hydrogen sulfide, P2X receptors

## Abstract

Lumbar disc herniation (LDH) is a major cause of discogenic low back pain and sciatica, but the underlying mechanisms remain largely unknown. Hydrogen sulfide (H_2_S) is becoming recognized for its involvement in a wide variety of processes including inflammation and nociception. The present study was designed to investigate the roles of the H_2_S signaling pathway in the regulation of expression and function of purinergic receptors (P2XRs) in dorsal root ganglion (DRG) neurons from rats with LDH. LDH was induced by implantation of autologous nucleus pulposus (NP), harvested from rat tail, in lumbar 5 and 6 spinal nerve roots. Implantation of autologous NP induced persistent pain hypersensitivity, which was partially reversed by an intrathecal injection of A317491, a potent inhibitor of P2X3Rs and P2X2/3Rs. The NP induced persistent pain hypersensitivity was associated with the increased expression of P2X3Rs, but not P2X1Rs and P2X2Rs, receptors in L5-6 DRGs. NP implantation also produced a 2-fold increase in ATP-induced intracellular calcium signals in DRG neurons when compared to those of controls (P < 0.05). Interestingly, NP implantation significantly enhanced expression of the endogenous hydrogen sulfide producing enzyme, cystathionine-β-synthetase (CBS). Systematic administration of *O*-(Carboxymethyl) hydroxylamine hemihydrochloride (AOAA), an inhibitor of CBS, suppressed the upregulation of P2X3R expression and the potentiation of ATP-induced intracellular calcium signals in DRG neurons (P < 0.05). Intrathecal injection of AOAA markedly attenuated NP induced- persistent pain hypersensitivity. Our results suggest that sensitization of P2X3Rs, which is likely mediated by CBS-H_2_S signaling in primary sensory neurons, contributes to discogenic pain. Targeting CBS/H_2_S-P2X3R signaling may represent a potential treatment for neuropathic pain caused by LDH.

## Introduction

Lumbar disc herniation (LDH) is one of the most common causes of discogenic low back pain and sciatica in clinical settings. Symptoms in patients are induced by both mechanical compression and chemical inflammation of the nerve roots. It is presumed that first-order sensory neurons in the associated dorsal root ganglia (DRGs) are affected by mechanical and chemical injury. Inflammatory reactions between nucleus pulposus (NP) and the nerve roots have been suggested to play an important role in disc herniation with sciatica [1-5]. Experimental studies have demonstrated that epidural application of NP leads to pronounced morphologic and functional changes in the nerve roots [6-9]. However, the pathogenic mechanisms linking herniated NP, gene expression, and pain hypersensitivity are not well understood.

Purinergic P2X receptors (P2XRs), which are ligand-gated cation channels, are preferentially expressed in DRG neurons and have been implicated in inflammatory activity [10], visceral pain hypersensitivity [11] and neuropathic pain [12-14]. Emerging evidence has suggested that the P2X3R plays an important role in immune responses and inflammatory diseases. Recently, many studies have confirmed that this receptor is also involved in the development of neuropathic pain [15-18]. Recent reports have shown an increase in P2X3R expression in primary sensory afferents [19,20]. In addition, local application of nucleus pulposus induces expression of P2X3Rs in rat dorsal root ganglion cells [9], suggesting a role for P2X3Rs in disc herniation and sciatica. However, the mechanism underlying P2X3R upregulation under LDH conditions remains largely unknown.

Hydrogen sulfide (H2S), synthesized by the endogenous enzymes cystathionine-β-synthetase (CBS) and cystathionine-γ-lyase (CSE), is increasingly recognized as a biologically important signaling molecule in various tissues and pathophysiological processes, including pain and inflammation [21-24]. Its putative role as a neurotransmitter/modulator is supported by recent reports on its effects on hippocampal neurons as well as peripheral sensory neurons [24-27]. With respect to the latter, intraplantar injection of NaHS (a commonly used H_2_S donor) in rat hindpaws produces mechanical hyperalgesia through activation of T-type Ca^2+^ channels, supporting a pro-nociceptive role for H_2_S [25]. H2S generation is enhanced in formalin [26] and carrageenan [28] models of persistent inflammatory pain. Colonic administration of H2S enhances pain behaviors in response to CRD in mice [22] and rats [17]. A growing body of evidence indicates a role for the CBS-H_2_S signaling pathway in inflammatory and neuropathic pain conditions. However, the role of CBS-H_2_S signaling in discogenic neuropathic pain hypersensitivity is unknown.

Our aim was therefore to study the potential role of H_2_S in the pathogenesis of sciatica hyperalgesia in a well-characterized rat model of lumbar disc herniation. In particular, we investigated whether the CBS-H_2_S and P2X3R signaling pathways were involved in discogenic neuropathic pain. We hypothesized that P2X3 receptors activated by the CBS-H_2_S signaling pathway participate in discogenic mechanical allodynia. To test this hypothesis, we investigated the roles of CBS and P2X3Rs in DRGs in LDH rats and a sham group of rats. Our results indicate that NP-induced peripheral discogenic pain hypersensitivity is likely mediated by upregulation of P2X3R expression in DRGs, and that CBS produces pronociceptive effects via activation of the CBS-H_2_S-P2X3R signaling pathway. These results may enhance our understanding of pathophysiological mechanisms associated with disc herniation and sciatica.

## Methods and materials

### Animals

**Figure 1:**
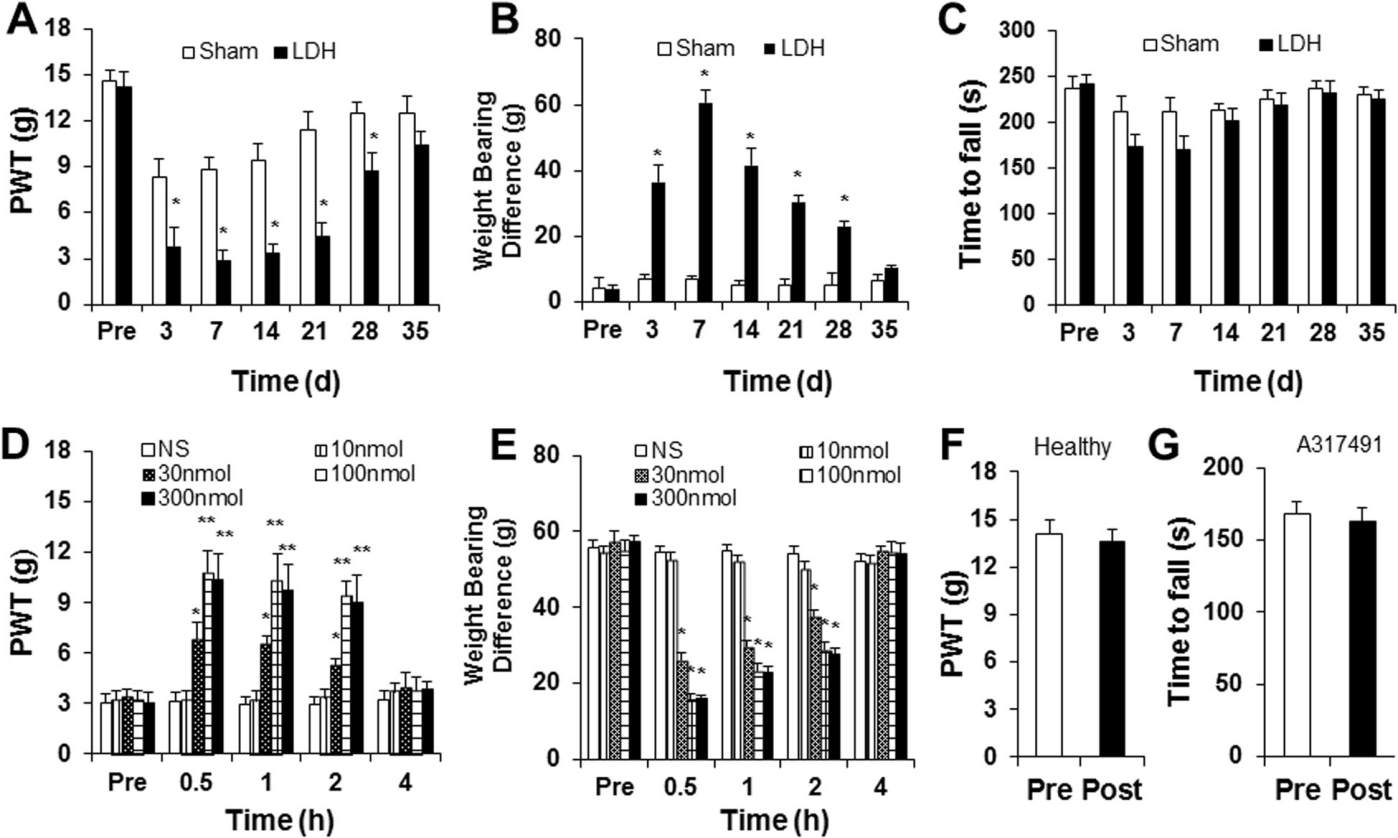
**Antagonism of P2X3 receptors on discogenic pain behavior. (A)** In rats treated with NP, the mechanical paw withdrawal threshold (PWT) was decreased significantly on the ipsilateral side at 3, 7, 14, 21 and 28 days after surgery compared to the sham group (*P < 0.05, n = 11 for the LDH group and n = 8 for the sham group). **(B)** In rats treated with NP, the body weight bearing difference was dramatically increased between hindlimbs at 3, 7, 14, 21 and 28 days after surgery compared to the sham group (*P < 0.05, n = 7 for the LDH group and n = 6 for the sham group). **(C)** Compared to the sham group, NP-application did not produce any effect on time to fall for rats on the Rota rod (n = 8 for each group, P > 0.05). **(D)** Administration of the P2X3R inhibitor A317491 mitigated mechanical hyperalgesia in LDH rats. The A317491-induced antinociceptive effects were observed at 30 min and disappeared 4 hours after a single intrathecal (it) injection of A317491 at the doses of 30,100 and 300 nmol. n = 6 rats for each group, *P < 0.05, **P < 0.01 compared to the NS group. **(E)** Administration of the P2X3R inhibitor A317491 reduced the body weight bearing difference induced by LDH. The A317491-induced antinociceptive effect disappeared 4 hours after a single intrathecal (it) injection of A317491 at doses of 30,100 and 300 nmol; n = 6 rats for each group *P < 0.05, compared to Pre injection values. **(F)** Administration of A317491 (it) did not produce any effect on PWT in healthy control rats (n = 5, P > 0.05). **(G)** Administration of A317491 (it) did not produce any effect on the time to fall for LDH rats in the Rota rod test (n = 8).

The Institutional Animal Care and Use Committee of Soochow University specifically approved this study. Experiments were performed on adult male Sprague–Dawley rats (220 ± 20 g). Animals were housed under controlled conditions (07:00 ~ 19:00 lighting, 24 ± 2°C) with free access to a standard laboratory diet and fresh water. Care and handling of rats were provided to ameliorate suffering in accordance with the guidelines of the International Association for the Study of Pain. In the present study, most experiments were performed 7 days after NP application, unless otherwise indicated. We selected this time point to perform experiments because the paw withdraw threshold was at the lowest point on the time-response curve (Figure 1A) and also to minimize the suffering of rats from pain hypersensitivity.

### Disc herniation model

Surgeries for the disc herniation model were performed as described in detail previously [22,29,30]. In brief, animals were divided into NP-treated and sham groups. All experimental procedures were performed on rats that were deeply anesthetized with sodium pentobarbital (50 mg/kg body weight, intraperitoneally). Additional doses of the anesthetics were administered as needed. The hair of rat’s lower back was shaved, and the skin was sterilized with 0.5% chlorhexidine and covered with clean paper. Sterile operating instruments were used. NP was harvested from the disc level between the second and third coccygeal intervertebral disc of each tail. To expose the lumbar 5 and lumbar 6 nerve roots, a midline dorsal incision from L4 to S1 was made over the lumbar spine. Harvested NP (approximately 5 mg) was implanted next to the left L5 and L6 nerve roots just proximal to the corresponding DRG. The amount of NP was applied approximately equally among rats. The right side of the dorsal roots was left intact without surgery in all rats. Special care was taken to minimize the mechanical compression at the time of surgery and prevent infection and minimize the influence of inflammation. The surgical procedure for the sham control group was identical to the NP treated group, including harvesting autologous NP from each rat tail and exposing nerve roots, but implantation of the autologous NP was omitted. After surgery, the rats were housed in individual cages in the animal room until they fully recovered.

### Pain behavior test

Rats (NP-treated group, n = 8, sham group n = 8) were tested for mechanical sensitivity of the plantar surface of the hindpaws 3 days before surgery and 3, 7, 14, 21, 28 and 35 days after surgery by an investigator blinded to the experimental group and protocol.

### Mechanical allodynia

Changes in hind paw mechanical withdrawal thresholds (PWT) were assessed utilizing von Frey filaments (VFF) as previously described [27,31-33]. A series of calibrated von Frey filaments (0.55, 0.93, 1.61, 1.98, 2.74, 4.87, 7.37, 11.42, 15.76, and 20.30) were applied perpendicularly to the plantar surface of the rat hind paw with sufficient force to bend the filaments for 60 s or until the rat withdrew. In the presence of a response, a filament of the next greater force was applied. In the absence of a response, a filament of the next lower force was applied. To avoid the potential injury, the cutoff strength of von Frey filaments was set at 20.30 g. The tactile stimulus producing a 50% likelihood of withdrawal was determined by the “up-down” calculating method, as described in detail previously [27,31-33]. Each test was repeated 2–3 times at approximately 2-min intervals, and the average value of VFF force was used as the force to evoke a withdrawal response.

### Body weight-bearing by the hind limbs

Hindlimb body weight-bearing differences were measured using an incapacitance tester (PH-200, Taimeng Chengdu, CHN). Measurements were recorded as averages of three trials, with each trial measuring the weight over 5 s. Weight-bearing is presented as weight on the contralateral limb - weight on the ipsilateral limb and weight-bearing asymmetry is indicative of hyperalgesia. Rats were tested on days 0 and 3–35 post-implantation.

### Western blotting

The protocols for western blotting were described previously by us [33-36]. In brief, lumbar DRGs (L5-6) from the ipsilateral side of the spinal cord were quickly dissected out and frozen in liquid nitrogen. Protein extracted from ipsilateral (L5-6) DRGs of NP-treated and sham rats were prepared in MT-CelLytics mammalian tissue protein extraction reagent with 1 mM PIC (1:100 dilution of protease inhibitor cocktail, Biocolor BioScience & Technology Company, CHN). Twenty micrograms of proteins were fractionated on 10% polyacrylamide gels (Bio-Rad) and then transferred to polyvinyl difluoride (PVDF) membranes (Roche) at 200 mA for 2 hours at 4°C. After membranes were blocked for 2 hours in TBS (50 mM Tris-Base, 133 mM NaCl, pH = 7.4) and a 5% dilution of non-fat milk powder, they were incubated with primary antibodies (mouse anti-CBS, anti-CSE Abnova Taiwan, CHN at 1:1000 and rabbit anti-P2X3R at 1:1000, anti-P2X1R and anti-P2X2R at 1:1000 Alomone, Israel) for 2 hours in 1% milk and TBS at room temperature. After washing in TBST (0.5% Tween-20 in TBS), the PVDF membranes were incubated with HRP conjugated secondary antibodies (1:4000, MultiSciences Biotech Co., CHN) in TBS and 1% milk for 2 hours at room temperature. Bands were visualized using ECL (Biological Industries, CHN) and exposed to Kodak X-ray films. Membranes were subsequently stripped and re-probed for GAPDH (1:1000, Hangzhou Goodhere Biotechnology Co., CHN). Films were scanned and band intensities were determined using Optic Quant software (ImageJ, NIH). CBS, CSE, P2X1, P2X2 and P2X3 receptor data were expressed as values normalized to GAPDH levels.

### Immunofluorescence study

As described previously [36], rats were deeply anesthetized one week after DiI injection. Animals were then perfused transcardially with 150 ml PBS followed by 400 ml ice-cold 4% paraformaldehyde in PBS. DRGs (L5 and L6) were removed and postfixed for 1 hour in paraformaldehyde and cryoprotected overnight with 20% sucrose in PBS. To ensure that a neuron was counted only once, serial sections were placed on consecutive slides with at least 50 μm between sections on the same slide. For triple labeling, 10 μm sections were simultaneously incubated with P2X3R (1:1000) and CBS (1:200) antibodies and then incubated with Alexa Fluor 488 and 355. The negative control was employed by omitting the primary antibody. Sections were viewed with filter cubes appropriate for DiI (rhodamine filter), Alexa 488 and Alexa 355. Images were captured and analyzed using Metaview software as described in detail previously [36].

### Rota rod analysis

The Rota rod system (ZH-300, Zhenghua, Anhui Province, CHN) for locomotor assessment was used to measure the time period for an animal to maintain its balance on a moving cylinder [37,38]. Animals were first conditioned on a stationary rod for 30 s, and during this time, animals that fell off the cylinder were placed back on the Rota rod. Next, the animals were conditioned at a constant speed of 20 rpm for a period of 300 s. Animals that failed the first conditioning period were allowed two additional periods, and those that failed the third conditioning period were omitted from further testing. This assured that all animals in all treatment groups attained an analogous baseline. The baseline values were 290.7 ± 3.4 seconds (n = 8) and 293.5 ± 3.5 seconds (n = 8) for sham and LDH, respectively. The same basic conditioning methodology was employed in the NP and sham groups. Thirty minutes after the last conditioning, each animal was placed on the Rota rod and its latency until falling was determined and expressed in seconds (s).

### Cell retrograde labeling

The origin of the primary afferent innervation of the sciatic nerve was determined by retrograde tracing using 1,19-dioleyl-3,3,39,3-tetramethylindocarbocyanine methanesulfonate (DiI, Invitrogen, Carlsbad, CA). Experiments were performed on male SD rats (200 ~ 220 grams, n = 20) as described in detail previously [34,35,39]. In brief, animals were anesthetized with chloral hydrate (360 mg/kg). Then, DiI (25 mg in 0.5 ml methanol) was slowly injected in 10 μL volumes into the left hindpaw at five different points (2 μl in each point) using a microinjection syringe after NP application or sham operation. The syringe was left in place for an additional 2 min to prevent the DiI from leaking out along the injection track. One week later, lumbar L5-6 DRGs were dissected out for the calcium imaging study or immunofluorescence study.

### Dissociation of DRG neurons and calcium imaging

The isolation of DRG neurons was performed 7 days after NP application or sham operation as described in detail previously [27,36]. In brief, animals were sacrificed by cervical dislocation, followed by decapitation. DRGs (L5-6) were then left ipsilaterally dissected out and transferred to an ice-cold, oxygenated dissecting solution. The dissecting solution contained (in mM): 130 NaCl, 5 KCl, 2 KH_2_PO_4_, 1.5 CaCl_2_, 6 MgSO_4_, 10 glucose, and 10 HEPES, pH 7.2 and osmolarity: 305 mOsm. After removal of connective tissue, DRGs were incubated for 1 h at 34.5°C in the dissecting solution which contained collagenase D (3.0 ~ 3.2 mg/ml, Roche; Indianapolis, IN) and trypsin (1.5 mg/ml, Sigma; St. Louis, MO). The ganglia were then washed and transferred to the dissecting solution containing DNase (0.5 mg/ml, Sigma, St. Louis, MO). After repeated trituration through flame-polished glass pipettes, a single-cell suspension was subsequently obtained for calcium imaging studies.

DRG neurons were then loaded with fura-2 acetoxymethyl ester (2 μM; Invitrogen, Carlsbad, CA) for 30 min at 37°C in an atmosphere of 5% CO_2_. Fura-2 acetoxymethyl ester was dissolved in normal external solution to which bovine serum albumin (5 mg/ml; Sigma-Aldrich) was added to promote dye loading. Ca^2+^ imaging was performed as described previously [40]. Briefly, coverslips were placed on an inverted epifluorescence microscope (IX70; Olympus, Tokyo, Japan) and continuously superfused with normal external solution. Fura-2 was excited alternately with UV light at 340 and 380 nm, and the fluorescence emission was detected at 510 nm using a computer-controlled monochromator. Image pairs were acquired every 5 s to 30 s using illumination periods between 100 and 250 ms in duration. Wavelength selection and the timing of excitation and acquisition of images were controlled using the Metaflour program (Molecular Devices). Digital images were stored for off-line analysis.

Drugs (ATP) were dissolved in external solution from concentrated stock solutions and delivered via bath application using a gravity-driven application system. A stock solution of ATP (20 mM) was made in ultrapure water and further diluted in normal external solution to the final concentration (20 μM). Image analysis was performed using the Metawave program. The background was subtracted to minimize camera dark noise and tissue autofluorescence. An area of interest was drawn around each cell, and the average value of all pixels included in this area was used as one measurement. The ATP-induced intracellular calcium mobilization was expressed as the ratio of the fluorescence signal measured at 340 nm to the fluorescence signal measured at 380 nm. Amplitudes of peak [Ca^2+^]_i_ responses were computed as the difference between the peak value and the baseline value. To be considered an ATP-induced response, changes in [Ca^2+^]_i_ had to occur within 2 to 3 min after drug application, and the amplitudes had to exceed baseline by 2 times the standard deviation.

### Drug application

AOAA (an inhibitor of CBS), A317491 (an antagonist of the P2X3 receptor), and ATP were purchased from Sigma-Aldrich and were freshly prepared in 0.9% normal saline. AOAA was intrathecally injected once daily for 7 consecutive days for molecular expression experiments and behavioral tests. A317491 was administered by a single intrathecal injection for behavioral tests.

### Data analysis

Data are expressed as mean ± standard error. Statistical analyses were performed using OriginPro 8 (OriginLab, US) and Matlab softwares (Mathworks, US). Normality was verified for all data before analyses. Significance was determined by the use of a two sample *t*-test, Kruskal-Wallis ANOVA followed by Tukey’s post hoc test, one-way or two-way repeated-measures ANOVA followed by Tukey’s post hoc test, as appropriate. Significance was set at P < 0.05.

## Results

### Inhibition of the P2X3 receptor suppresses NP-induced pain hypersensitivity

Implantation of autologous nucleus pulposus (NP) harvested from the rat tail to the lumbar 5 and lumbar 6 spinal nerve roots produced pain hypersensitivity in rats when compared with sham rats. In the NP-treated group, mechanical paw withdrawal threshold (PWT) decreased significantly on the ipsilateral side 3 days after surgery, indicating ipsilateral mechanical allodynia. This change persisted through day 28, and returned to baseline level on day 35 after implantation (*P < 0.05, Figure 1A). Compared to the sham group, the NP-treated rats showed a dramatic increase in body weight bearing difference. The body weight bearing difference was increased on day 3 after NP application, and returned to baseline level on day 35 after NP application (*P < 0.05, Figure 1B). To determine whether surgery had an effect on motor performance, Rota rod tests were performed in this study. There was no significant difference in the time that rats remained on the bar rotating at a fixed-speed (20 rpm) between the LDH and sham groups (Figure 1C, P > 0.05).

A-317491 was used to determine whether P2X3 receptors are involved in the development of allodynia in NP-treated rats. A-317491 has been shown to have a high affinity and selectivity for blocking P2X3 homomeric and P2X2/3 heteromeric receptor channels and to produce antinociception in rat models of chronic inflammatory and neuropathic pain [41]. A single intrathecal injection of A317491 produced an antinociceptive effect on PWT and weight bearing in LDH rats (n = 8 for each group, Friedman ANOVA). Injection of A-317491 at doses of 10 nmol did not produce a significant effect on PWT (Figure 1D) and body weight bearing (Figure 1E). However, injection of A-317491 at 30,100 or 300 nmol markedly increased PWT and reduced the body weight bearing difference. The PWTs were markedly increased at 30 min, which lasted for 2 hours after the A-317491 injection, when compared to the NS group (Figure 1D, *P < 0.05, **P < 0.01, n = 8 for each group, Kruskal-Wallis ANOVA followed by Tukey’s post hoc test). The body weight bearing difference was also remarkably decreased at 30 min and lasted for 2 hours after A-317491 injection when compared to the NS group (Figure 1E, *P < 0.05, n = 8 for each group, Tukey’s post hoc test following Kruskal–Wallis ANOVA). In contrast, injection of A-317491 at a dose of 100 nmol did not produce any effect on PWTs in age-matched healthy control rats (n = 5, Figure 1F). The effect of A317491 on motor activity in the LDH rats was determined using the Rota rod test. There was no significant difference in the time that rats remained on the bar rotating at a fixed-speed (20 rpm) before (Pre) and after (Post) injection of A317491 (Figure 1G, paired sample *t*-test, P > 0.05, n = 8 rats).

### NP-application upregulates expression of P2X3 receptors

**Figure 2:**
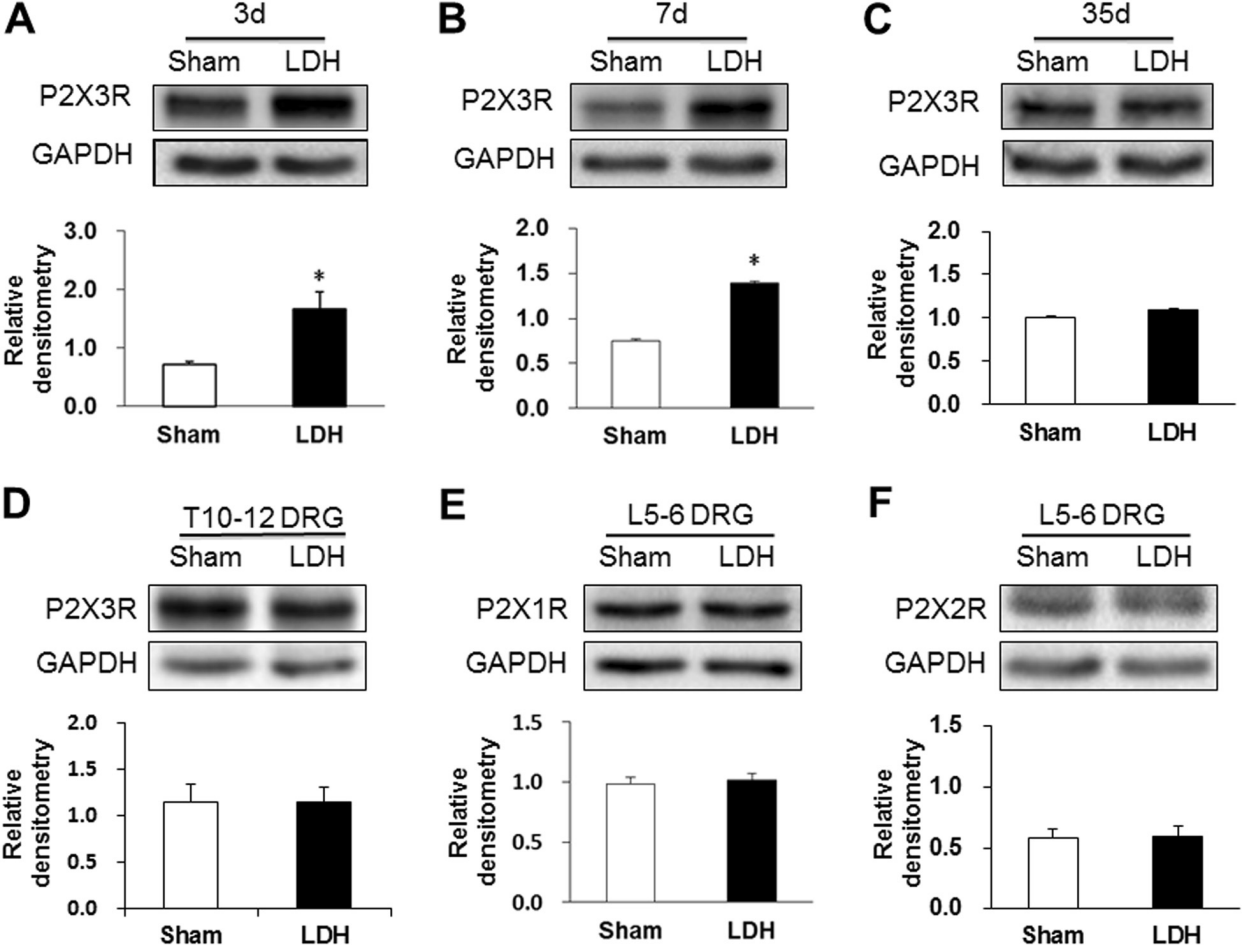
**Increased expression of P2X3 receptors in LDH rats. (A)** Expression of P2X3Rs in L5-6 DRGs was significantly enhanced 3 days after NP application when compared to the sham group (n = 6 for each group, *P < 0.05, two sample *t*-test). **(B)** Expression of P2X3Rs in L5-6 DRGs was remarkably enhanced 7 days after NP application when compared to the sham group (n = 6 for each group, *P < 0.05, two sample *t*-test). **(C)** Expression of P2X3Rs in L5-6 DRGs was not altered 35 days after NP application when compared to the sham group (n = 6 for each group, P > 0.05, two sample *t*-test). **(D)** There were no significant differences in P2X3R expression in T10-12 DRGs between the sham and LDH groups (n = 6 for each group). **(E)** NP application did not alter the expression of P2X1 receptors in L5-6 DRGs when compared to the sham group (n = 6 for each group, P > 0.05, two sample *t*-test). **(F)** NP application did not alter the expression of P2X2 receptors in L5-6 DRGs when compared to the sham group (n = 6 for each group, P > 0.05, two sample *t*-test). A GAPDH control for each sample was used as a loading control.

To determine the mechanism underlying the NP-induced mechanical allodynia, the expression levels of P2X3Rs in lumber DRGs were analyzed. Proteins were extracted from L5-6 DRGs of rats at 3, 7 and 35 days after NP-application or sham surgery. At 3 days after NP application, P2X3R protein expression was significantly increased after NP-treatment (Figure 2A, *P < 0.05, two sample *t*-test). The relative densitometry was 1.67 ± 0.30 (n = 6) in the NP-treated group and 0.72 ± 0.05 (n = 6) in the sham group. At 7 days after NP application, P2X3R protein expression was markedly increased after NP-treatment (Figure 2B, *P < 0.05, two sample *t*-test). The relative densitometry was 1.38 ± 0.20 (n = 6) in the NP-treated group and 0.75 ± 0.07 (n = 6) in the sham group. At 35 days after NP application, P2X3R protein expression was not altered after NP-treatment (Figure 2C, P > 0.05, two sample *t*-test). The relative densitometry was 1.09 ± 0.03 (n = 6) in the NP-treated group and 1.01 ± 0.04 (n = 6) in the sham group. To confirm the specificity, expression of P2X3 receptors in thoracic DRGs was determined. There were no significant differences in the expression of P2X3 receptors in T10-12 DRGs between the sham and LDH groups (Figure 2D, P > 0.05). The relative densitometry was 1.21 ± 0.13 (n = 6) in the NP-treated T10-T12 DRGs and 1.29 ± 0.15 (n = 6) in T10-T12 DRGs of the sham group. We also examined the expression of other subtypes of purinergic receptors. The relative densitometry of P2X1 receptors was 1.02 ± 0.06 (n = 6) in the NP-treated L5-6 DRGs and 0.98 ± 0.06 (n = 6) in L5-6 DRGs of the sham group. The relative densitometry of P2X2 receptor was 0.60 ± 0.08 (n = 6) in the NP-treated L5-6 DRGs and 0.58 ± 0.08 (n = 6) in L5-6 DRGs of the sham group. The expression of P2X1 and P2X2 receptors was not altered significantly 7 days after NP-treatment when compared to sham rats (Figure 2E and F, P > 0.05, two sample *t*-test).

### NP-application potentiates ATP-induced calcium signals

**Figure 3:**
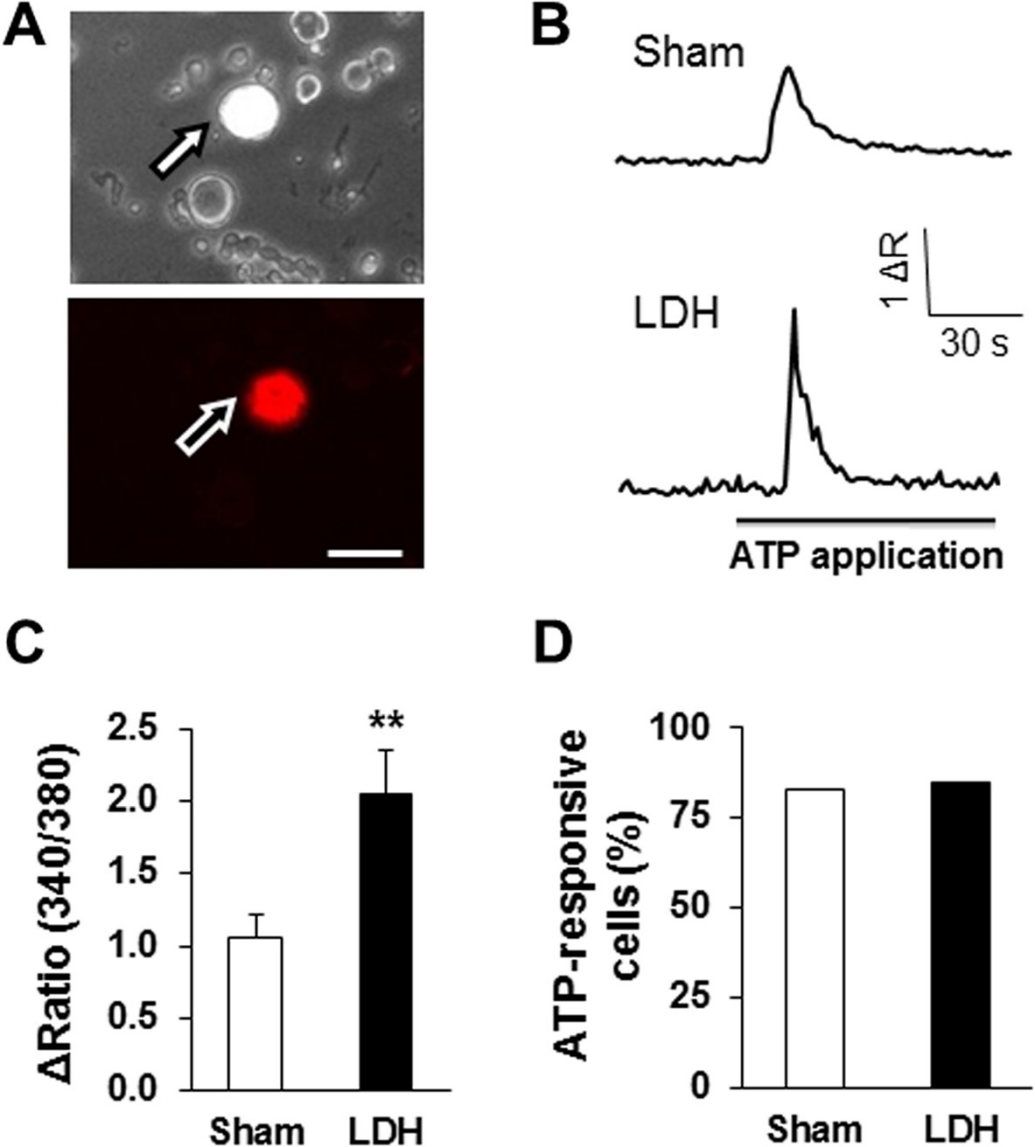
**Potentiation of ATP-induced intracellular calcium mobilization in LDH rats. (A)** Bright-field (upper) and DiI-fluorescence (lower) images of DRG neurons. A hindpaw innervating DRG neuron is shown in red (arrow). Bar = 50 μm. **(B)** An example trace of ATP (20 μM) -induced calcium signals in a DiI-labeled DRG neuron from a sham (upper) and LDH (lower) rat. **(C)** Bar graph showing a potentiation of ATP-induced Ca^2+^ transients in hindpaw innervating DRG neurons. NP application significantly enhanced ATP-induced Ca^2+^ transients of L5-6 DRG neurons when compared to the sham group (sham, n = 46 cells, LDH, n = 46 cells, *P < 0.05, two sample *t*-test). **(D)** The percentage of neurons responding to ATP application was not significantly altered in LDH rats when compared to sham rats (n = 80 cells for the sham group and n = 90 cells for the LDH group, P > 0.05, *χ* ^2^ test).

We next examined whether NP-application enhances ATP-induced responses in DRG neurons labeled by DiI. Only small and medium cells (25 to 35 μm in diameter), which are known to mediate the transmission of nociceptive signals [42], were used in the present study. We first examined the effect of NP-application on ATP-evoked calcium mobilization. As described above, L5-6 DRG neurons were labeled by DiI (Figure 3A, arrows). Application of ATP at a concentration of 20 μM elicited significantly larger calcium signals in NP treated animals than in sham rats (Figure 3B and C, n = 46 cells for each group). In contrast, the percentage of numbers of neurons responding to ATP application was not significantly altered in LDH rats when compared to sham rats (Figure 3D). The percentage of numbers of neurons was 82.5% and 84.4% for sham group and LDH group, respectively.

### Inhibition of CBS activity reverses expression and function of P2X3Rs

**Figure 4:**
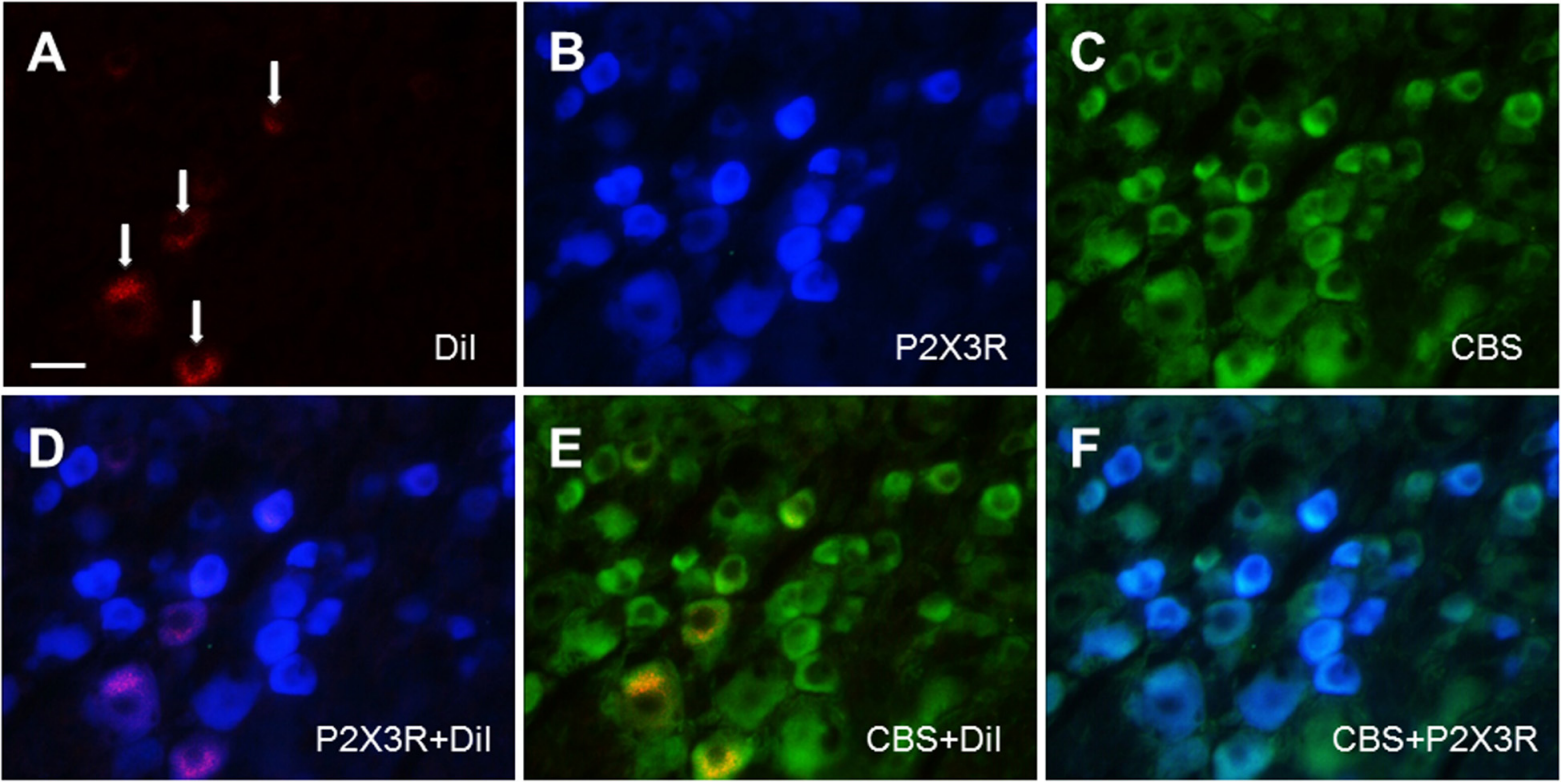
Co-expression of CBS with P2X3 receptors in hindpaw innervating DRG neurons. **(A)** L5 DRG cells innervating the hindpaw were labeled with DiI (red) injected into the hindpaw. **(B)** P2X3R positive cells are shown in blue. **(C)** CBS positive cells are shown in green. **(D)** Merge of double labeling of DiI and P2X3Rs in DRGs. **(E)** Merge of CBS-positive staining and DiI labeling. **(F)** Merge of CBS staining and P2X3R labeling. Bar = 50 μm.

We next determined the mechanisms underlying the sensitization of P2X3 receptors (P2X3Rs) under the LDH condition. Since we have previously shown that the endogenous H_2_S producing enzyme cystathionine-β-synthetase (CBS) plays a role in inflammatory pain and visceral pain conditions [11,17,34,43], we determined whether sensitization of P2X3Rs is regulated by endogenous H_2_S signaling. We first examined whether P2X3Rs were co-localized with CBS in DiI labeled DRG neurons. Triple-labeling techniques showed that 4 neurons (arrows) that were immunoreactive for P2X3 receptors were also positive for CBS (Figure 4). Similarly, 4 neurons that were immunoreactive for CBS were also positive for P2X3 receptors (Figure 4). These data indicate that P2X3Rs are co-expressed with CBS in hindpaw innervating DRG neurons.

**Figure 5:**
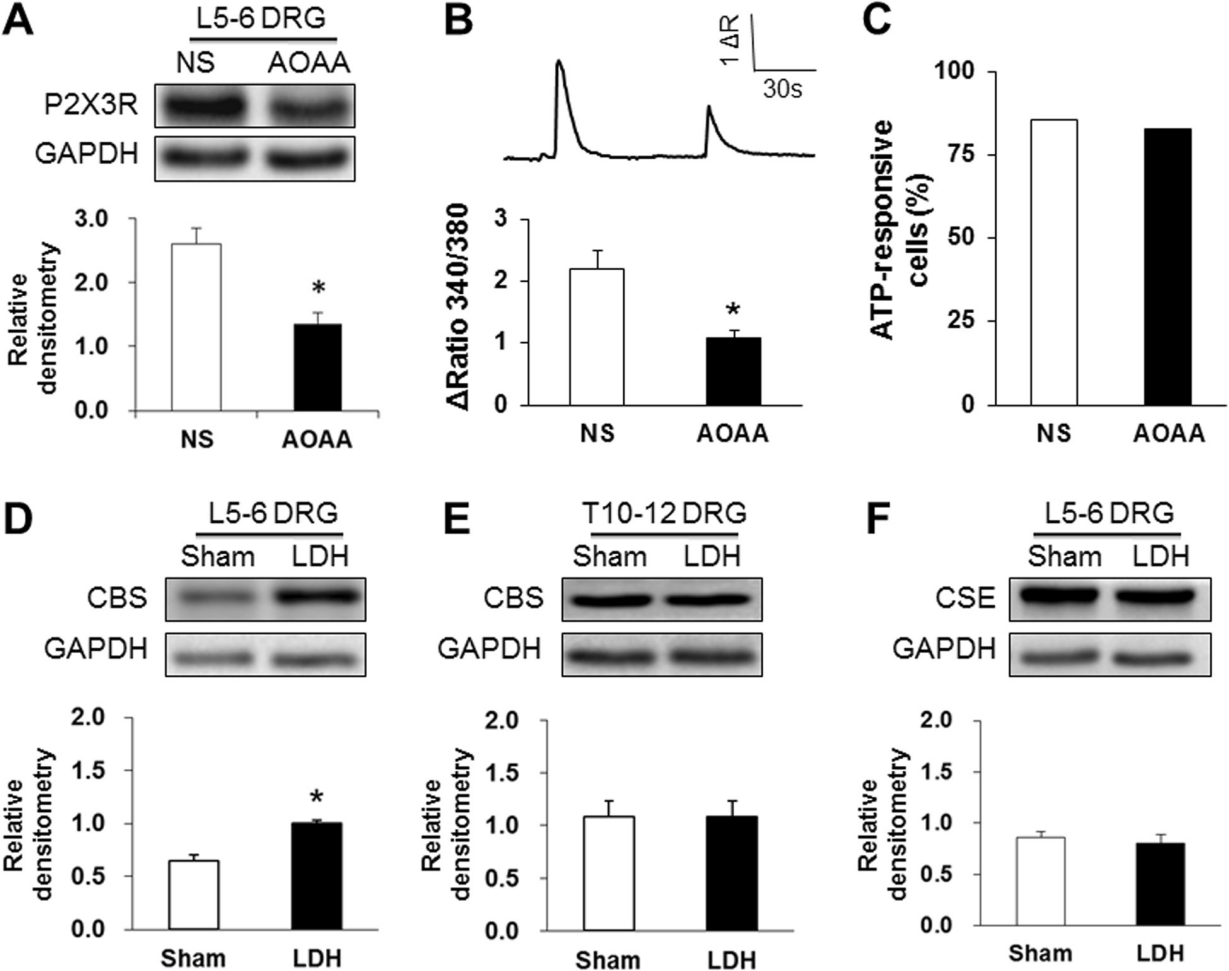
**Antagonism of the CBS inhibitor on the expression and function of P2X3 receptors. (A)** Administration of the CBS inhibitor O-(carboxymethyl) hydroxylamine hemihydrochloride (AOAA, 10 μg/kg) significantly reduced the expression of P2X3Rs in L5–L6 DRGs from LDH rats (*P < 0.05, n = 7 for each group). **(B)** In LDH rats, application of AOAA greatly reduced the ATP-evoked intracellular calcium mobilization when compared to NS-treated rats (NS, n = 48 cells, LDH, n = 56 cells, *P < 0.05, two sample *t*-test). **(C)** The percentage of neurons responding to ATP application was not significantly altered in LDH rats when compared to sham rats (n = 96 cells for each group, P > 0.05, *χ* ^2^ test). **(D)** LDH treatment significantly enhanced expression of CBS in L5–L6 DRGs when compared to sham rats (n = 7 for each group, *P < 0.05). **(E)** There was no significant difference in CBS expression in T10-12 DRGs between the sham and LDH groups (n = 6 for each group). **(F)** There was no significant difference in CSE expression in L5-6 DRGs between the sham and LDH groups (n = 6 for each group).

P2X3 receptor expression was then measured after treatment with AOAA. AOAA at 10 μg/kg body weight (once every day for 7 consecutive days) was used since this dose significantly attenuated the pain behavior. AOAA intrathecal injection remarkably reversed the expression of P2X3R in DRGs from LDH rats when compared to the NS group. The relative densitometry of P2X3R was 2.60 ± 0.24 (n = 7) for the NS group and 1.35 ± 0.17 (n = 7) for the AOAA group (Figure 5A, *P < 0.05, two sample *t*-test). In addition, AOAA treatment significantly reduced the ATP-evoked intracellular calcium mobilization when compared with NS treatment in LDH rats (Figure 5B, *P < 0.05, n = 48 cells for the NS group, n = 56 cells for the AOAA group, two sample *t*-test). In contrast, the percentage of neurons responding to ATP application was not significantly altered in AOAA-treated rats when compared with NS-treated rats (Figure 5C).

Furthermore, expression of CBS in L5-6 DRGs was examined after NP application. NP application markedly increased the expression of CBS when compared to the sham group. The relative densitometry was 1.00 ± 0.03 (n = 7) in the NP-treated group and 0.65 ± 0.05 (n = 7) in the sham group (Figure 5D, *P < 0.05, two sample *t*-test). There were no significant differences in CBS expression in T10-12 DRGs between the sham and LDH groups. The relative densitometry was 1.09 ± 0.15 (n = 6) in the NP-treated group and 1.09 ± 0.15 (n = 6) in the sham group (Figure 5E, P > 0.05, two sample *t*-test). We also investigated the expression of cystathionine-γ-lyase (CSE), another endogenous H_2_S producing enzyme. The expression of CSE in L5-6 DRGs was not altered significantly 7 days after NP application when compared to sham rats. The relative densitometry was 0.81 ± 0.09 (n = 6) in the NP-treated group and 0.86 ± 0.06 (n = 6) in the sham group (Figure 5F, P > 0.05, two sample *t*-test).

### Intrathecal administration of CBS inhibitor attenuates NP-induced pain hypersensitivity

**Figure 6:**
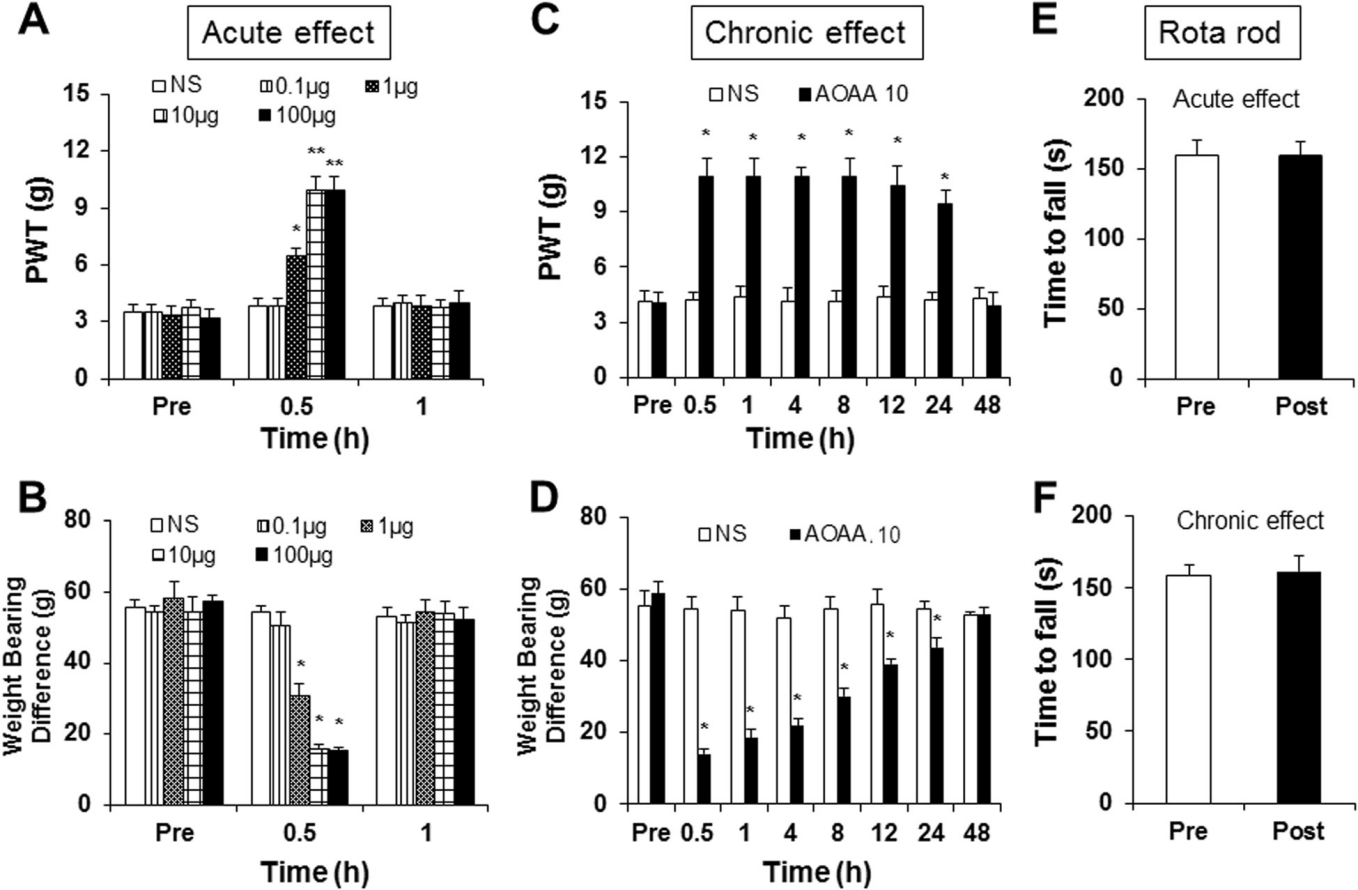
**Antagonism of intrathecal injection of CBS inhibitor on LDH-induced pain hypersensitivity. (A)** A single intrathecal injection of the CBS inhibitor AOAA attenuated mechanical allodynia induced by LDH. The maximal effect occurred at a dose of 10 μg/kg body weight in the present study. The AOAA-induced antinociceptive effect dissipated 1 hour after injection. n = 8 for each group, *P < 0.05 compared to Pre injection values. **(B)** A single intrathecal injection of the CBS inhibitor AOAA reduced the body weight bearing difference induced by LDH. The maximal effect occurred at a dose of 10 μg/kg body weight. The AOAA-induced antinociceptive effect dissipated 1 hour after injection. n = 6 for each group, *P < 0.05 compared to NS. **(C)** Chronic effects of AOAA injection (10 μg/kg, once every day for 7 consecutive days) on mechanical withdrawal threshold. Multiple injections of AOAA attenuated PWT, which lasted for 24 hours after injection. *P < 0.05 versus NS, n = 8 for each group. **(D)** Multiple injections of AOAA reduced the body weight bearing difference, which lasted for 24 hours after injection. *P < 0.05 versus NS, n = 7 for each group. **(E)** A single intrathecal injection of AOAA did not produce any effect on the time that rats remained on the bar during the Rota rod test. **(F)** Multiple intrathecal injections of AOAA did not produce any effect on the time that rats remained on the bar during the Rota rod test.

We then determined whether CBS is involved in the development of pain hypersensitivity in LDH rats. We observed the acute and chronic effects of the CBS inhibitor AOAA on PWT and body weight bearing difference in LDH rats. One day after NP application, a total of 26 LDH rats were intrathecally administered AOAA at different doses (0.1, 1.0, 10.0 and 100.0 μg/kg body weight) in a volume of 10 μl. PWT and body weight bearing differences were recorded from LDH rats 30 min after a single injection of AOAA. NS and AOAA at the lower doses (0.1 μg/kg) had no significant effect on the PWT and body weight bearing tests. However, AOAA at 1, 10 and 100 μg/kg produced a significant increase in PWT (Figure 6A, n = 6, 6, 7, 7 and 5 for the NS group and AOAA was administered at 0.1, 1.0, 10.0 and 100.0 μg/kg, respectively, **P < 0.01, compared to the NS group, Kruskal-Wallis ANOVA followed by Tukey’s post hoc test). The antinociceptive effect returned to baseline level at 1 hour after injection of AOAA. Similarly, a single injection of AOAA significantly reduced the body weight bearing difference in LDH rats, starting 30 min after injection and returning to baseline level one hour after injection (Figure 6B, n = 6 for each group, *P < 0.01, compared to the NS group; Kruskal-Wallis ANOVA followed by Tukey’s post hoc test). To further determine the antinociceptive effect of the CBS inhibitor, AOAA at 10 μg/kg was administered intrathecally once daily for 7 consecutive days. PWTs and body weight bearing tests were performed 30 min after the last injection of AOAA and continued to be measured for 48 hours. As expected, multiple injections of AOAA at 10 μg/kg produced a dramatic antinociceptive effect on both the PWT and body weight bearing difference, lasting for at least 24 hours (Figure 6C and D, n = 7 rats for each group, *P < 0.01, Friedman ANOVA). Single or multiple injections of AOAA at 10 μg/kg did not produce a significant effect on the time that rats remained on the Rota rod bar when compared with Pre injection performance (Figure 6E and F, n = 8 rats for each group, P > 0.05).

### Hindpaw administration of CBS inhibitor did not alter NP-induced pain hypersensitivity and the expression of P2X3 receptors

**Figure 7:**
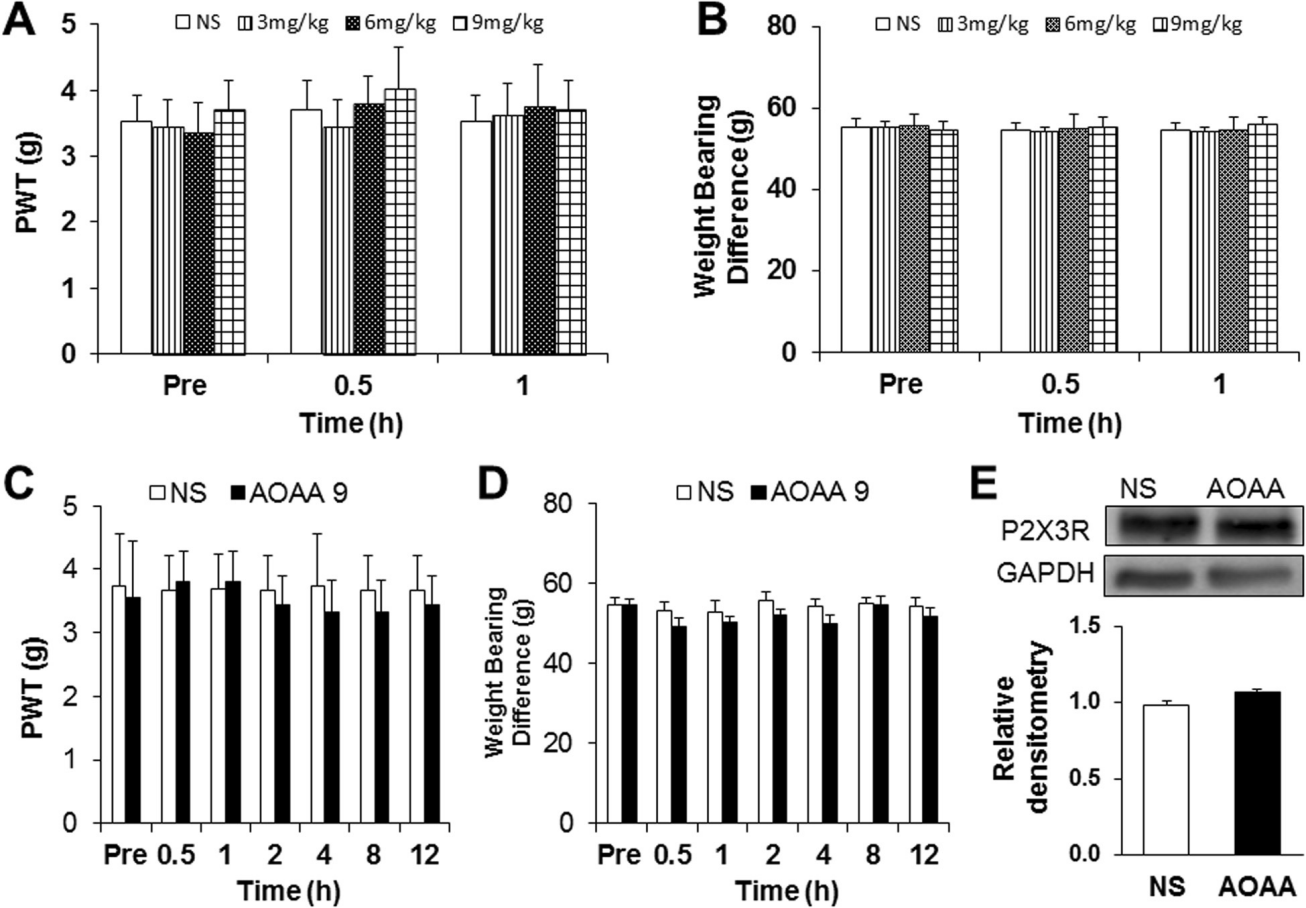
**Antagonism of hindpaw injection of CBS inhibitor on LDH-induced pain hypersensitivity. (A)** A single hindpaw injection of the CBS inhibitor AOAA at different doses (3, 6 and 9 mg/kg body weight) did not significantly alter mechanical allodynia induced by LDH. **(B)** A single hindpaw injection of the CBS inhibitor AOAA did not alter the body weight bearing difference induced by LDH. **(C)** Chronic effects of AOAA injection (9 mg/kg body weight, once every day for 7 consecutive days) did not altered mechanical withdrawal threshold. **(D)** Multiple injections of AOAA did not alter the body weight bearing difference. **(E)** Multiple hindpaw injections of AOAA did not affect protein levels of P2X3 receptor of LDH rats (n = 4 for each group).

We then determined whether peripheral CBS is involved in the development of pain hypersensitivity in LDH rats. We observed the acute and chronic effects of AOAA injected into the hindpaw on PWT and body weight bearing difference in LDH rats. As shown in Figure 7, neither a single injection (Figure 7A and B, n = 6 for each group) nor multiple injection (Figure 7C and D, n = 6 for each group) of AOAA produced significant effects on PWT (Figure 7A and C) and BWD (Figure 7B and D) of LDH rats. In addition, multiple injection of AOAA into hindpaw did not significantly alter the expression of P2X3 receptors of LDH rats (Figure 7E, n = 4). These data suggest that CBS at peripheral tissues might not play a role in the development of LDH induced pain hypersensitivity.

## Discussion

The present study demonstrated that autologous NP application to the spinal nerve produced a prolonged mechanical allodynia accompanied by reduced body weight bearing by the affected limb, suggesting that it was painful to place full weight on the hindpaw. In addition, we showed for the first time that these behaviors were correlated with increased expression of P2X3 receptor proteins and the endogenous hydrogen sulfide producing enzyme CBS in the corresponding DRGs. Importantly, spinal inhibition of P2X3Rs by A317491 or CBS by AOAA during the maintenance phase attenuated NP-induced discogenic pain hypersensitivity. However, hindpaw injection of AOAA did not alter the pain hypersensitivity in LDH rats. These results suggest that CBS-H_2_S and purinergic signaling pathways at spinal level are involved in NP-induced peripheral discogenic pain hypersensitivity in rats.

The intrathecal injection route is traditionally believed to target both the spinal cells and the DRG cells [37,38,44-47]. Here, intrathecal injection of a P2X3R antagonist may inhibit the P2X3 receptors in both the spinal cord and DRGs. In parallel with the increased expression of P2X3 receptors in DRGs, our behavioral data showed that inhibition of the P2X3R by intrathecal injection of A317491 markedly enhanced the paw withdrawal threshold and reduced the body weight bearing difference at 7 days after NP application. Additionally, it was reported that local or systematic administration of a P2X3R antagonist reversed the mechanical allodynia in rats treated with streptozotocin- [14] or cancer cells [48-50]. Inhibition of P2X receptors also attenuated the visceral hypersensitivity induced by neonatal colonic inflammation [11], suggesting that P2X receptors contribute to the maintenance of the various chronic pain conditions. Of note is that the prolonged duration of AOAA effect observed in the present study would be due to the central effect since the intrathecally administered drugs might have wide access to the central nervous system shortly after administration. This needs to be further investigated.

The upregulation of P2X3R expression in L5 and L6 DRGs is consistent with our previous report [51] and supported by calcium imaging studies showing that NP application enhanced ATP-induced intracellular calcium mobilization of DRG neurons innervating the hindpaw. In the present study, we also observed that the expression level of P2X3Rs was well correlated with changes in pain hypersensitivity in rats after NP application. However, P2X1 and P2X2 receptor expression was not altered after NP application. In addition, expression of P2X3 receptors was not altered in thoracic DRGs (e.g., T10-12) after NP application. These data suggest a specific role for P2X3Rs in a rat model of LDH in terms of expression, and that upregulation of P2X3Rs in corresponding DRGs is not a non-specific effect after NP application. Purinergic P2XR activation has been shown in physiological [52] and various pathophysiological pain conditions, including inflammatory pain [10,53], neuropathic pain [54,55], visceral pain [11] and cancer pain [48,50]. However, the molecular mechanisms underlying the upregulation of P2C receptors remain largely unknown.

In the present study, we showed that P2X3Rs was colocalized with CBS in hindpaw innervating DRG neurons, suggesting a possible interaction between P2X3Rs and CBS in DRG neurons. The results of western blot analysis further confirmed that an increase in P2X3R expression was regulated by CBS activity in the DRGs, since inhibition of CBS activity reversed the upregulation of P2X3Rs. This evidence supports that P2X3Rs and CBS may be involved in neuropathic pain at DRG levels. Importantly, CBS upregulation was only observed in L5 and L6 DRGs rather than in nearby DRGs (e.g., T10 ~ 12 DRGs). In addition, expression of CSE, another H_2_S producing enzyme, was not changed in the DRGs after NP application. Taken together, these results support the idea that CBS may play an important role in the regulation of P2X3R expression and function under pathophysiological conditions. It has been demonstrated that hydrogen sulfide (H_2_S) can regulate neuronal excitability by regulating voltage-gated sodium channels [35,36,56], voltage-gated potassium channels [39] and voltage-gated calcium channels [57], thus contributing to the development of chronic pain hypersensitivity. Here, we added evidence to show that P2X3R expression and function is regulated by CBS activity. Although we only examined CBS expression in the DRGs, other modulators, such as proinflammatory cytokines, chemokines, and nerve growth factors, may also increase and contribute to NP-induced pain hypersensitivity. In addition, the detailed mechanisms underlying the regulation of P2X3R activities by CBS activation need to be further investigated.

In summary, the present study demonstrates for the first time that the expression of P2X3Rs and CBS is significantly enhanced in DRGs after NP application, and that intrathecal administration of a CBS antagonist remarkably attenuates the pain hypersensitivity in a rat model of LDH. Our findings suggest that the increased CBS expression may mediate NP-induced pathological changes by increasing P2X3R expression and function in the DRG neurons. The targeting of CBS/H_2_S signaling may be a potential treatment strategy for chronic radicular neuropathic pain.
